# Upregulation of phosphoserine phosphatase contributes to tumor progression and predicts poor prognosis in non‐small cell lung cancer patients

**DOI:** 10.1111/1759-7714.13064

**Published:** 2019-04-11

**Authors:** Li Liao, Huajian Yu, Mengxi Ge, Qiong Zhan, Ruofan Huang, Xiaoyu Ji, Xiaohua Liang, Xinli Zhou

**Affiliations:** ^1^ Department of Oncology Huashan Hospital Fudan University Shanghai China; ^2^ State Key Laboratory of Oncogenes and Related Genes, Shanghai Cancer Institute, Renji Hospital Shanghai Jiao Tong University School of Medicine Shanghai China

**Keywords:** AKT/AMPK signaling pathway, metastasis, NSCLC, proliferation, PSPH

## Abstract

**Background:**

Growing evidence indicates that high phosphoserine phosphatase (PSPH) expression is associated with tumor prognosis in many types of cancers. However, the role of PSPH in non‐small cell lung cancer (NSCLC) is unclear. The purpose of this study was to investigate the clinical significance of PSPH in NSCLC.

**Methods:**

One hundred forty‐three patients with histologically confirmed NSCLC who underwent surgery were included. Quantitative real‐time PCR and Western blot were used to assess PSPH expression in paired tumor and corresponding adjacent non‐tumorous tissues. The role of PSPH in invasion and cell growth was investigated in vitro.

**Results:**

Compared to adjacent normal lung tissues, PSPH messenger RNA and protein levels were significantly higher in NSCLC tissues, and the PSPH expression level was positively related to clinical stage, metastasis, and recurrence. High PSPH expression was predictive of poor overall survival. A549 cells transfected with small interfering‐PSPH showed inhibited cell migration, invasion, and proliferation. We further demonstrated that PSPH might promote the invasive capabilities of NSCLC cells through the AKT/AMPK signaling pathway.

**Conclusion:**

Our results indicate that PSPH may act as a putative oncogene in NSCLC, and may be a vital molecular marker for the metastasis and proliferation of NSCLC cells by regulating the AKT/AMPK signaling pathway.

## Introduction

Lung cancer is the most lethal solid tumor and the leading cause of cancer‐related death worldwide,^1^, ^2^ with both median follow‐up and overall survival (OS) rates in all patients of > 14 months after initial diagnosis.^3^ Non‐small cell lung cancer (NSCLC) is the predominant type of lung cancer and accounts for approximately 85% of cases. Despite significant clinical improvements in systematic treatment, approximately 40–60% of stage I and II NSCLC patients die of distant metastases, which is a critical contributor to the mortality rate.^4^, ^5^ Furthermore, the molecular mechanisms underlying lung cancer metastasis are still largely unclear. Therefore, exploring effective markers for the diagnosis and prognosis of NSCLC, as well as potential therapeutic targets, is clinically valuable.^6^

Phosphoserine phosphatase (PSPH) belongs to a subfamily of phosphotransferases. PSPH is located in chromosome 7p11.2, which encodes enzymes, and was originally known as a mediator involved in the third and last step in L‐serine formation.^7^ Previous studies have reported that PSPH mainly participates in multiple fundamental aspects of cell behavior by providing amino acid, neurotransmitters, and nucleotide substrates necessary for cell proliferation and division.^8^, ^9^ Additionally, PSPH participates in neuronal signal transduction and methylation.^10^ Thus, deficiency of this protein is thought to be linked to Williams syndrome and neural tube defects.

PSPH also acts as a positive regulator and is involved in the malignancy of a variety of cancers, including phyllodes tumor**,** 11 breast cancer,^12^ hepatocellular carcinoma,^13^ and Ewing sarcoma,^14^ because the *PSPH* gene is stimulated during tumorigenesis and metastasis, which leads to the production of greater amounts of L‐serine.^15^ Although accumulating literature suggests that PSPH may be a critical regulator in the progression of human cancers, the role of PSPH dysregulation and its underlying molecular mechanism in NSCLC progression has not been explored. In this research, we investigated the prognostic effect of PSPH in NSCLC and determined the role of PSPH in NSCLC cell proliferation and metastasis.

## Methods

## Clinical human non‐small cell lung cancer (NSCLC) tissues

The Ethical Review Committee of Huashan Hospital approved the study and all patients provided informed consent. All human NSCLC tissue and their matched normal adjacent tissue samples (at least 3 cm from the primary tumor) were obtained from 143 NSCLC patients who underwent surgery at Huashan Hospital, Fudan University (Shanghai, China) between 2014 and 2018. All human surgical specimens and lymph node metastases were pathologically diagnosed at Huashan Hospital.

## Cell culture

The human A549 NSCLC cell line used in this study was purchased from American Type Culture Collection (ATCC, Rockville, MD, USA). As described in previous reports, A549 cells were cultured in RPMI‐1640 medium (HyClone, Logan, UT, USA) containing 10% fetal bovine serum (FBS; Biowest, South America Origin, Riverside, MO, USA) and routinely cultivated in a humidified air atmosphere incubator at 37°C with 5% carbon dioxide.

## Cell proliferation

Cell proliferation was determined using Cell Counting Kit 8 (CCK‐8) assay (Dojindo, Kumamoto, Japan) according to the manufacturer's instructions. NSCLC cells were seeded in triplicate wells of 96‐well plates at 1 × 10^3 cells per well in a final volume of 200 μl, and 10 μl of CCK‐8 solution was then added into 100 μl fresh RPMI‐1640 in each well and incubated for two hours at 37°C. The growth curve was prepared according to the absorbance of each well at 450 nm. Experiments were performed independently three times.

## Clone formation assays

To evaluate the ability to form sizable colonies, 1 × 10^3 cells were seeded in six‐well plates after transfection with small interfering RNA (siRNA) for 48 hours. The plates were then incubated at 37°C for seven days until cells had formed sufficiently large clones. At the end of the experiments, the cells were washed three times with phosphate buffered saline (PBS), fixed with 100% methanol for 30 minutes, and stained with 0.1% crystal violet for 10 minutes. The cells were washed thoroughly with tap water and air‐dried. Finally, the number of visible colonies was counted by light microscopy. The assays were performed independently three times.

## Cell cycle analysis

The distribution of cell cycle stages was analyzed using flow cytometry. Cells were cultured in six‐well plates, harvested with trypsinization, and washed twice with ice‐cold PBS. The cells were then fixed with 70% ethanol diluted in PBS at −20°C overnight. After washing with PBS, the cells were collected by centrifugation at 1000 rpm for 5 minutes, resuspended, and stained with propidium iodide (Beyotime, Beijing, China) in the dark at 37 °C for 30 minutes according to the manufacturer's instructions. The percentage of the cell cycle phase completed was analyzed using a FACSCalibur flow cytometer (BD Biosciences, San Jose, CA, USA). The assays were performed independently three times.

## Cell migration and invasion assays

Cell migration and invasion assays were performed using 8 μm pore size Transwell filter chamber inserts (Corning, Christiansburg, VA, USA). For migration assays, 5 × 10^4 cells suspended in 200 μl RPMI‐1640 without FBS medium were added into the upper chamber. Inserts were then placed in 24‐well plates with 800 μl RPMI‐1640 containing 10% FBS. For invasion assays, Matrigel (BD Biosciences) was diluted to 1 mg/mL with RPMI‐1640 without FBS medium and immediately added to the upper chamber. After hydrating for three or four hours, 1 × 10^5 cells suspended in 200 μl RPMI‐1640 without FBS medium were placed into the upper chamber and inserts were placed in 24‐well plates with 800 μl RPMI‐1640 containing 10% FBS. After incubation for 14 and 20 hours at 37°C and 5% CO_2_, respectively, cells that had moved to the bottom (below the filter surface) of the membrane were fixed with 100% methanol for 30 minutes, stained with 0.1% crystal violet for 10 minutes, and washed with water three times. The invading cells were then counted and imaged in at least five random fields under a light microscope (Olympus Corporation, Tokyo Japan). The assays were performed independently three times.

## Wound‐healing assays

Cells were plated in six‐well plates at approximately 80% confluence. Four separate wounds were carefully made by scraping a 200 ml sterile pipette tip across the confluent cell monolayer. The cell debris was gently washed and removed with PBS and the remaining cells were incubated with 2 ml fresh RPMI‐1640 media supplemented with 1% FBS. Five selected fields at the lesion border were then photographed at 0 (control), 24, and 48 hours, and the migrated areas were measured. All samples were assayed in triplicate.

## RNA extraction and quantitative real‐time PCR analysis

Total RNA samples of A549 cell lysates and tissues were extracted in accordance with TRIzol reagent (Invitrogen, Carlsbad, CA, USA). Complementary DNA was synthesized from 2 μg of each RNA sample and reverse transcribed using the PrimeScript RT Reagent Kit (TaKaRa, Shiga, Japan) according to the manufacturer's instructions. Quantitative real‐time (qRT) PCR was subsequently conducted using the SYBR Green Premix Ex Taq kit (TaKaRa). β‐actin was used as the internal reference gene for quantifying messenger RNA (mRNA) levels. The primer sequences are shown in Table 1.

**Table 1:** Primer sequences used in quantitative real‐time PCR analysis

mRNA	Forward primer	Reverse primer
PSPH	5’‐CACGGTCATCAGAGAAGAAG‐3’	5’‐GGTTGCTCTGCTATGAGTCT‐3’
β‐actin	5′‐ TGTGGCCGAGGACTTTGATT −3’	5′‐ CCTGTGTGGACTTGGGAGAG −3’

mRNA, messenger RNA, PSPH, phosphoserine phosphatase.

## Protein extraction

Cell protein was lysed on ice using a mixture of T‐PER Protein Extraction Reagent lysates (Thermo Fisher Scientific, Waltham, MA, USA) plus protease and phosphatase inhibitors (Yeasen, Guangzhou, China) according to the manufacturer's instructions. After 30 minutes incubation, the protein concentration was determined using a Bicinchoninic Acid Protein Assay Kit (Pierce, Biotechnology, Rockford, IL, USA).

## Western blot analysis

Protein samples (20 μg) were separated using 10% sodium dodecyl sulfate‐polyacrylamide gel electrophoresis, transferred to polyvinylidene difluoride membranes (Millipore, Billerica, MA, USA), and blocked with 5% non‐fat dried milk for one hour. The membranes were then incubated with mouse anti‐PSPH (1:200), mouse anti‐p‐FAK (1:200), and rabbit anti‐FAK (1:200, Santa Cruz Biotechnology Inc., Santa Cruz, CA, USA); rabbit anti‐p‐mTOR (1:1000), rabbit anti‐mTOR (1:1000), rabbit anti‐p‐Stat3 (1:1000), rabbit anti‐Stat3 (1:1000), rabbit anti‐p‐AKT (1:1000), rabbit anti‐AKT (1:1000), rabbit anti‐p‐AMPK (1:1000), rabbit anti‐AMPK (1:1000), rabbit anti‐p‐JNK (1:500), and rabbit anti‐JNK (1:500, Cell Signaling Technology, Danvers, MA, USA) overnight at 4 °C. The next day, the membranes were incubated with horseradish peroxidase conjugated anti‐mouse immunoglobulin G1 (1:5000) and horseradish peroxidase conjugated anti‐rabbit immunoglobulin G (1:5000, Sigma‐Aldrich, St. Louis, MO, USA) for two hours. The membranes were then washed three times with PBS with Tween 20 between each antibody incubation step. Finally, the proteins on membranes were visualized using a LumiBest ECL Reagent Solution Kit (Share‐bio, Shanghai, China). β‐actin was used as the loading control.

## RNA interference using small interfering RNA

Transfections were performed to decrease PSPH expression using siRNA. Two siRNA oligonucleotides targeted at PSPH were designed and synthesized by RiboBio (Guangzhou, China). The target sequences were as follows: si‐PSPH#1: 3′‐GGAGCGAAATGTTCAGGTT‐5′; si‐PSPH#2: 3′‐GGCAACAAGTCAAGGATAA‐5′; si‐NC was used as the control. Transient transfection was performed using Lipofectamine 2000 Reagents (Invitrogen) according to the manufacturer's protocols. After 48 hours, the transfection efficiency was monitored using qRT‐PCR and Western blotting.

## Bioinformatic analysis

The Gene Expression Omnibus (GEO, https://www.ncbi.nlm.nih.gov/geo/), an RNA and DNA microarray sequence database, was employed to assess the correlation between the PSPH expression level and NSCLC clinicopathological features using a Student's *t*‐test. Data of PSPH expression in lung cancer was obtained from the UALCAN database (http://ualcan.path.uab.edu/index.html) using links to The Cancer Genome Atlas (TCGA). The survival curve was analyzed in CaArray and GEO dataset GSE30219 using the Kaplan–Meier Plotter (http://kmplot.com/analysis/).

## Statistical analysis

The data are presented as the mean ± standard error of the mean from one representative experiment out of three independent experiments, unless stated otherwise. The comparisons of quantitative results were analyzed by Student's *t*‐test. *P* < 0.05 was considered statistically significant.

## Results

### Patient characteristics

A total of 143 paired NSCLC samples with full clinicopathological data were investigated. The clinicopathologic characteristics are shown in Table 2. The majority of patients were male (87 cases, 60.8%), with adenocarcinoma (104 cases, 72.7%), and without metastasis (84 cases, 58.7%). The mean age was 62.53±9.30 (range: 35–81) years. Pathological results showed that 54 of 143 patients (37.76%) had local lymph node metastasis. Only 12 patients (8.39%) in the cohort had distant metastasis (4 brain, 8 bone). The mean maximum diameter of primary NSCLC tumors was 3.50 ± 1.89 cm (range: 1.0–13.5 cm).

**Table 2:** Relationship between PSPH expression and clinicopathologic characteristics in 143 NSCLC patients

Characteristics	Number of cases (%)	PSPH expression	
		Mean ± SD	*P*
Age			
≤ 60	57 (39.8)	0.00214 ± 0.00363	0.168
>60	86 (60.2)	0.00346 ± 0.00650	
Gender			
Female	56 (39.2)	0.00193 ± 0.00232	0.086
Male	87 (60.8)	0.00358 ± 0.00682	
Tumor size (cm)			
≤ 3	55 (38.5)	0.00229 ± 0.00531	0.279
>3	88 (61.5)	0.00334 ± 0.00569	
Tissue			***
NSCLC	143	0.00296 ± 0.00557	**0.000**
Noncancerous	143	0.00113 ± 0.00151	
Pathological type			
Adenocarcinoma	104 (72.7)	0.00265 ± 0.00485	0.324
Squamous cell carcinoma	39 (27.3)	0.00369 ± 0.00710	
Degree of differentiation			
Well and moderately	30 (21)	0.00313 ± 0.00492	0.836
Poorly	113 (79)	0.00289 ± 0.00573	
Clinical stage			***
I + II	91 (63.6)	0.00168 ± 0.00230	**0.000**
III + IV	52 (36.4)	0.00513 ± 0.00828	
Metastasis			**
No	84 (58.7)	0.00164 ± 0.00298	**0.010**
Yes	59 (41.3)	0.00436 ± 0.00769	

* *P* < 0.05,

** *P* < 0.0,1 and

*** *P* < 0.001;

bold text indicates significance. Comparisons of quantitative results were analyzed by Student's *t* test. NSCLC, non‐small cell lung cancer; PSPH, phosphoserine phosphatase; SD, standard deviation.

### Evaluation of phosphoserine phosphatase (PSPH) expression in NSCLC

**Figure 1:**
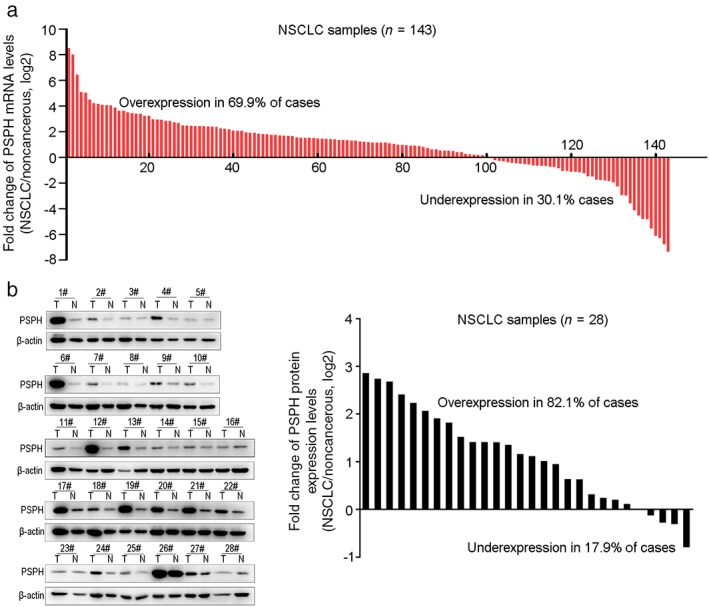
Relative expression of phosphoserine phosphatase (PSPH) in non‐small cell lung cancer (NSCLC) patients. (**a**) The PSPH expression levels in 143 paired non‐small cell lung cancer (NSCLC) and corresponding non‐cancerous tissues were measured by quantitative real‐time PCR. (**b**) Western blot analysis of 28 paired NSCLC and corresponding non‐cancerous tissues. mRNA, messenger RNA.

To determine the potential clinical implications of PSPH expression in NSCLC progression, we examined the relative expression of PSPH mRNA in NSCLC (*n* = 143) and corresponding non‐tumorous tissues (*n* = 143) using qRT‐PCR. The PSPH mRNA expression level was statistically higher in NSCLC tissues than in adjacent normal tissues (*P* = 2.8E^‐05). High PSPH expression was found in 69.9% (100/143) of NSCLC cases. In contrast, only 30.1% (43/143) of the cases exhibited enhanced PSPH expression in the adjacent normal tissues (Fig 1a).

**Figure 2:**
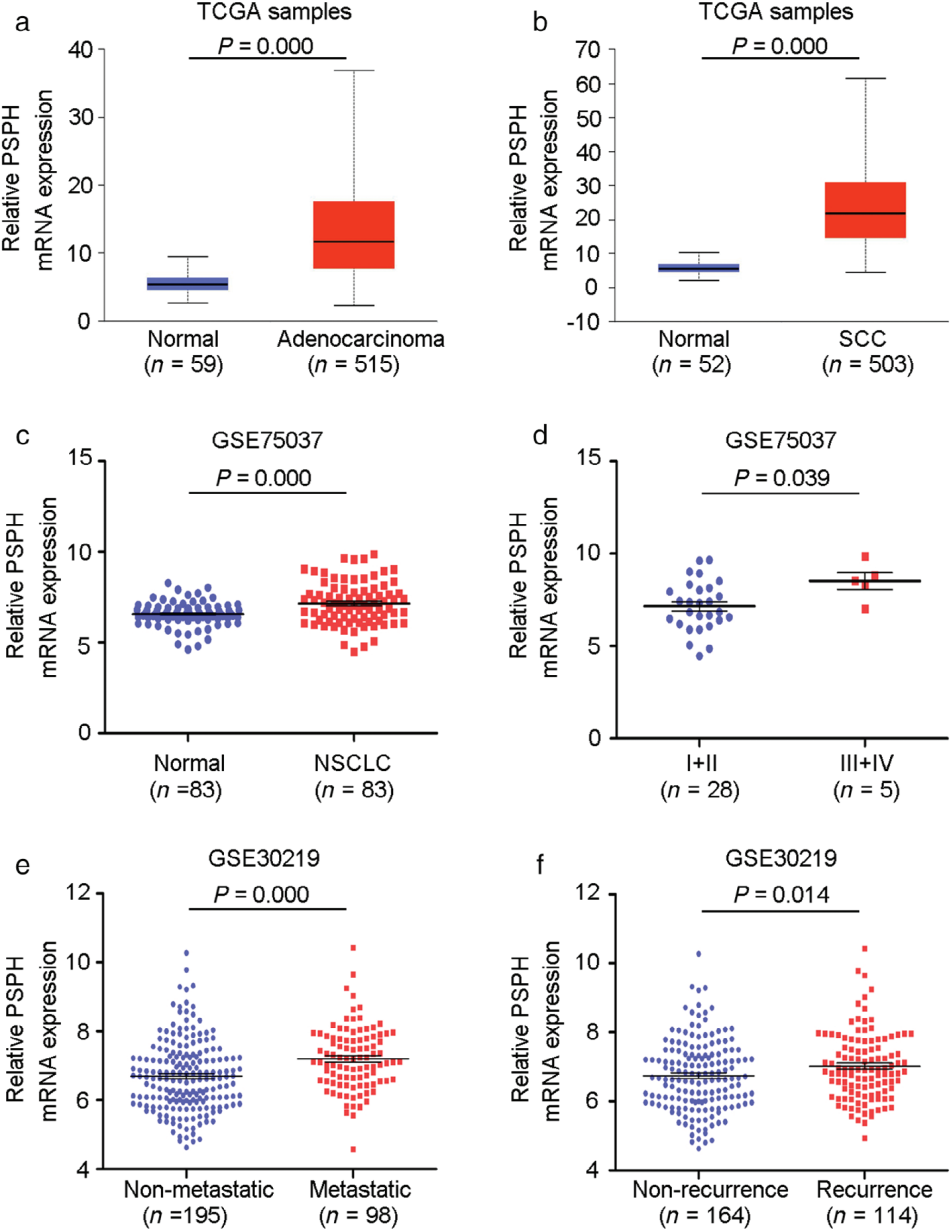
Evaluation of phosphoserine phosphatase (PSPH) expression in non‐small cell lung cancer (NSCLC) through bioinformatic analysis. (**a**,**b**) PSPH was overexpressed in adenocarcinoma and squamous cell carcinoma (SCC) samples from The Cancer Genome Atlas (TCGA) database. (**c**) PSPH expression was assessed in 83 paired NSCLC tissues from the Gene Expression Omnibus. (**d**,**e**) The upregulation of PSPH messenger RNA (mRNA) in NSCLC was positively associated with metastasis (*P* = 0.000) and recurrence (*P* = 0.014).

To further confirm the results of qRT‐PCR, we analyzed PSPH protein expression in 28 NSCLC and corresponding adjacent non‐tumorous samples using Western blot. PSPH protein was significantly overexpressed in NSCLC tissues compared to the matched adjacent normal lung tissues (*P* = 0.025) (Fig 1b). Further analysis showed that the PSPH protein level was upregulated in 82.1% (23/28) of NSCLC tissues. Moreover, we also analyzed TCGA and GEO data to evaluate PSPH expression in NSCLC patients. Consistent with our data, PSPH was significantly overexpressed across the two datasets (*P* = 0.000 in both adenocarcinoma and squamous cell carcinoma) (Fig 2a,b). Additionally, PSPH expression was significantly elevated in NSCLC compared to normal lung tissues from GSE75037 (*P* = 0.000) (Fig 2c). Thus, these results indicate that PSPH is highly expressed in NSCLC tissues.

### Relationship between PSPH expression and clinicopathological characteristics of NSCLC patients

We analyzed the relationship between PSPH expression and the pathological data of the 143 NSCLC cases. The PSPH expression level was significantly associated with clinicopathological features, including lymph node and distal metastasis (*P* = 0.010) and advanced tumor node metastasis (TNM) stages (*P* = 0.0003), but not with age, gender, pathological type, differentiation, or tumor size. GEO high‐throughput data was also used to assess the relationship between PSPH expression and clinicopathological characteristics in NSCLC. The results showed that the PSPH expression level was strongly associated with metastasis (*P* = 0.000) (Fig 2d) and recurrence (*P* = 0.014) (Fig 2e). Thus, we can speculate that PSPH may have a pivotal role in promoting aggressive phenotypes in NSCLC.

### Overexpression of PSPH indicates poor prognosis in NSCLC

**Figure 3:**
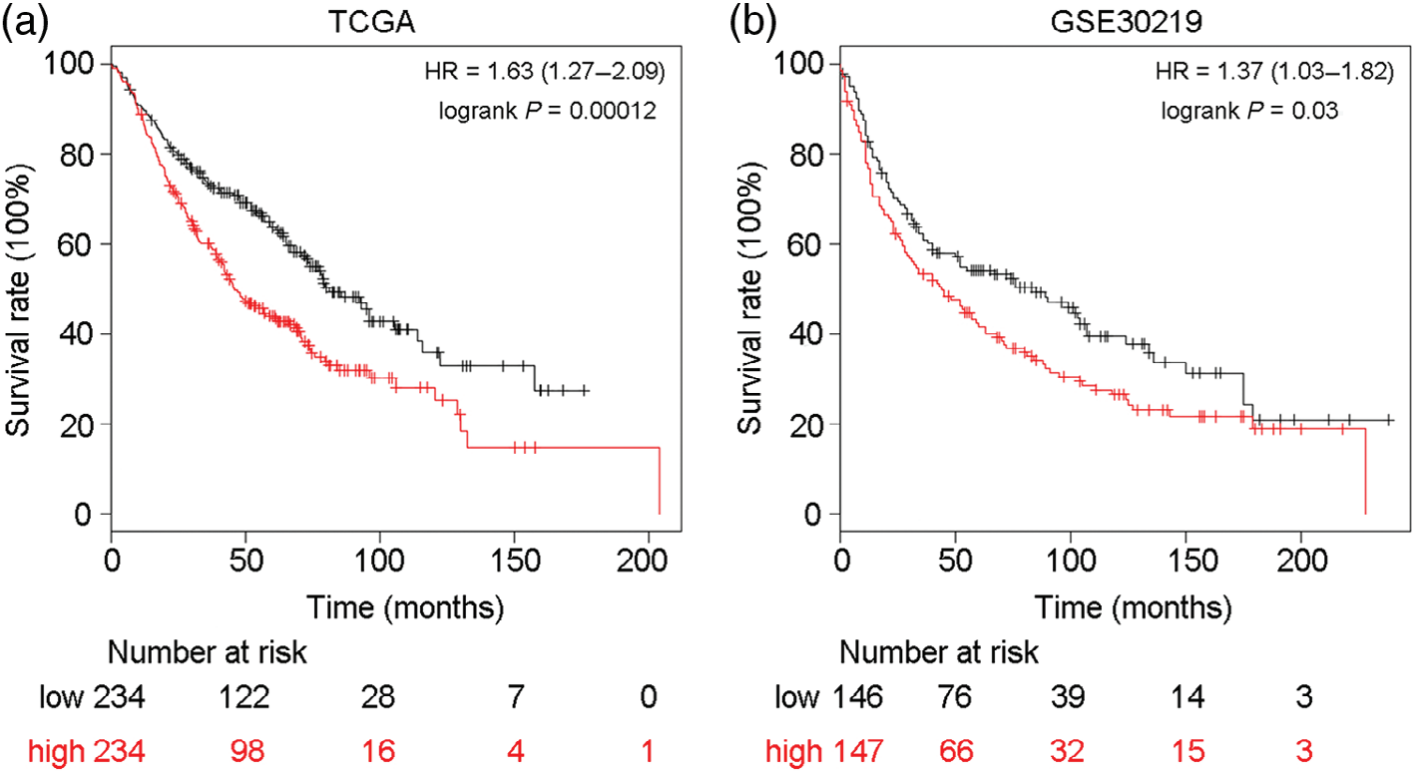
The Kaplan–Meier Plotter was used to evaluate survival in non‐small cell lung cancer (NSCLC) patients. (**a**,**b**) Patients with high phosphoserine phosphatase (PSPH) expression had worse overall survival than those with low PSPH expression (*P* < 0.05). (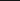) PSPH low expression and (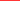) PSPH high expression.

To evaluate whether PSPH has prognostic value in NSCLC patients, we analyzed TCGA and GEO data to evaluate the relationship between OS and PSPH expression level. Statistical analyses of survival suggested that patients with high PSPH expression had significantly poorer OS than those with low PSPH expression (Fig 3). Therefore, PSPH may serve as a potential predictor for assessing poor prognosis in NSCLC.

### PSPH enhances the proliferation of NSCLC cells during the G2/M phase

**Figure 4:**
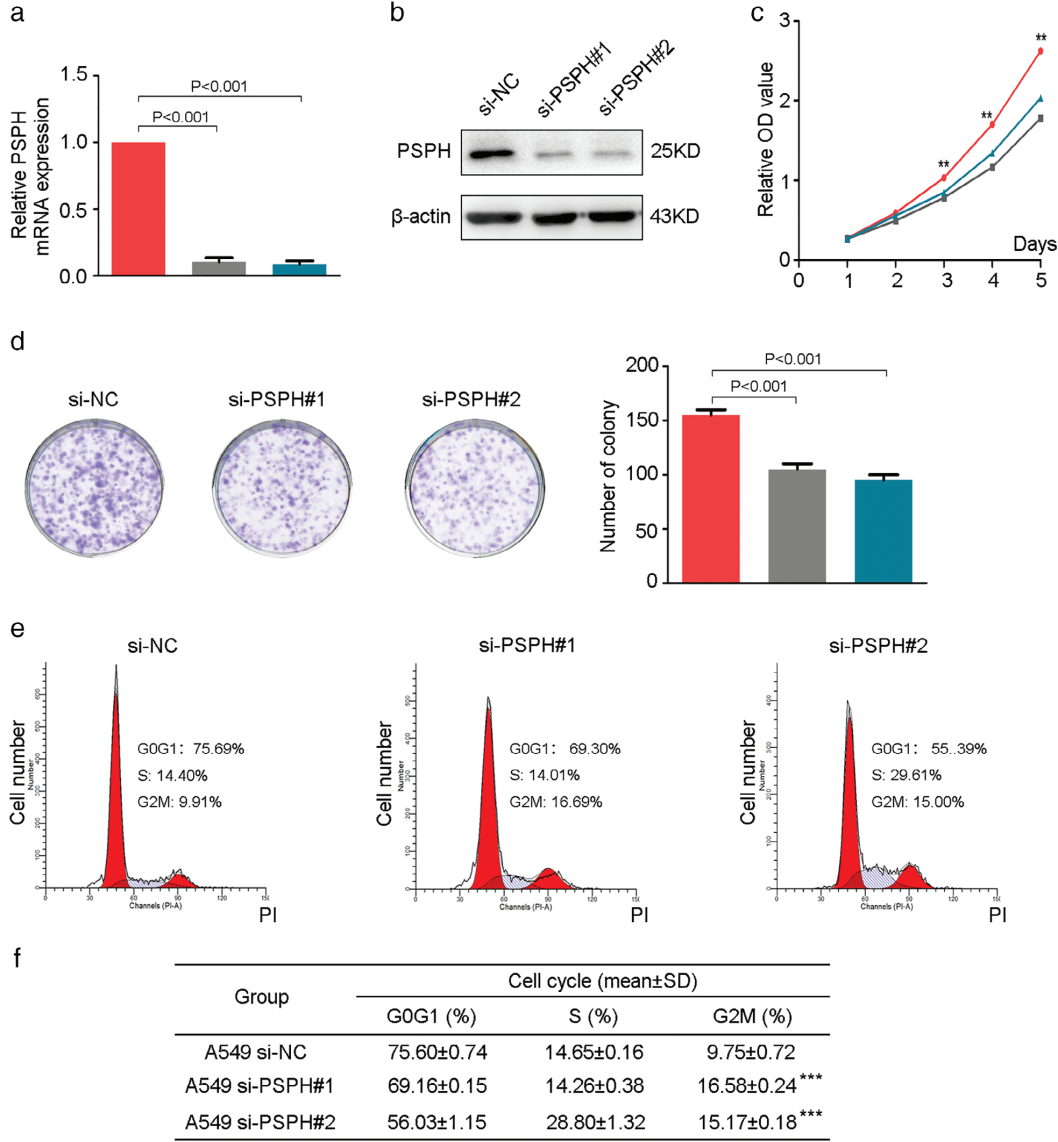
Phosphoserine phosphatase (PSPH) knockdown inhibits cell proliferation by arresting the G2/M cell cycle. (**a**,**b**) Determination of transfection efficiency using quantitative real‐time PCR and Western blot. (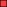) si‐NC, (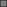) si‐PSPH#1, and (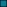) si‐PSPH#2. (**c**) Diagrams showing results of Cell Counting Kit‐8 assays. A549 cell proliferation was inhibited by downregulation of PSPH expression. (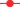) si‐NC, (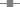) si‐PSPH#1, and (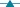) si‐PSPH#2. (**d**) Representative photomicrographs of A549 cell colonies in culture plates and significant reduction in the colony formation efficacy in A549 cells following PSPH knockdown. (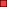) si‐NC, (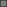) si‐PSPH#1, and (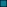) si‐PSPH#2. (**e**,**f**) Flow cytometric analysis showing the percentages of PSPH knockdown cells at different phases of the cell cycle. (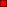) G0G1, (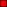) G2M, and (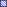) S. Data are expressed as the mean ± standard deviation. ***P < 0.001 by Student's *t*‐test. OD, optical density; siNC, small interfering negative control.

As unlimited growth, invasion, and metastasis are hallmarks of tumor malignancy, we explored the role of PSPH in human NSCLC progression using a transient transfection strategy. Knockdown efficiency was determined by qRT‐PCR and Western blot. A549 cells transfected with si‐PSPH had significantly lower expression of PSPH compared to negative controls at both the transcription and protein levels (Fig 4a,b). We examined the effect of PSPH on cell proliferation using CCK‐8 and colony forming assays. The results demonstrated that PSPH silencing significantly suppressed the proliferation and reduced the number of colonies of A549 cells (Fig 4c,d). These results indicate that PSPH may promote NSCLC cell proliferation.

To gain deeper insight into the effect of PSPH on cell growth, we examined the cell cycle profile of the si‐NC and si‐PSPH groups using flow cytometry analysis. Cell cycle analysis revealed G2/M phase arrest in A549 cells after the deletion of PSPH (Fig 4e,f). Thus, we can conclude that the depletion of PSPH significantly inhibits cell proliferation by inducing cell cycle arrest in the G2/M phase.

### PSPH promotes the migration and invasion ability of NSCLC cells

**Figure 5:**
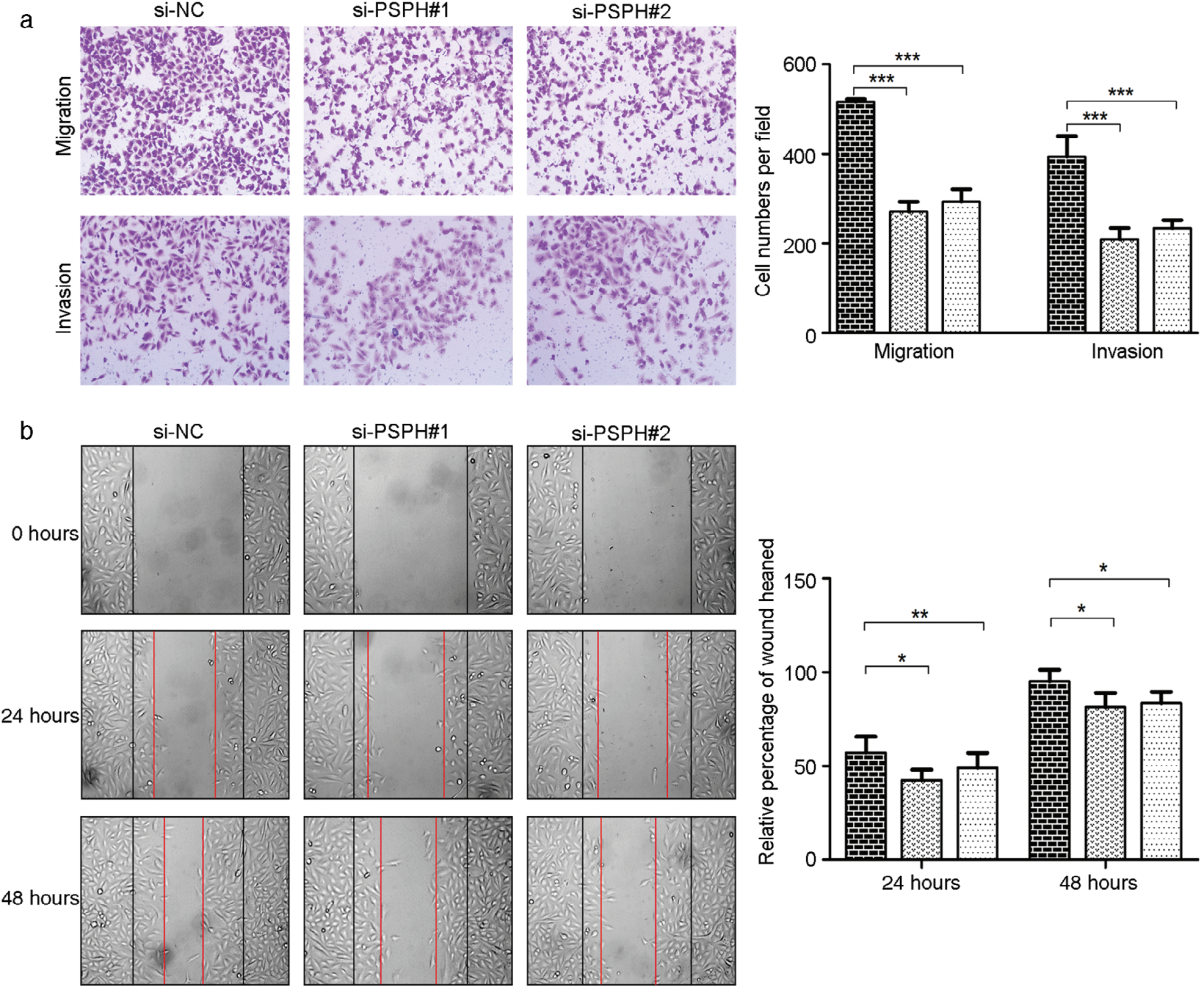
Depletion of phosphoserine phosphatase (PSPH) reduces cell migration and invasion in non‐small cell lung cancer (NSCLC) cells. (**a**) Transwell migration and invasion and (**b**) wound‐healing assays of A549 were conducted after transduction with the small interfering negative control (si‐NC) or si‐PSPH. Error bars represent standard error of the mean. (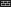) si‐NC, (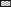) si‐PSPH#1, and (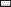) si‐PSPH#2. *P < 0.05, **P < 0.01, and ***P < 0.001 by Student's *t*‐test.

Transwell and wound‐healing assays were performed to evaluate cell migration and invasion capabilities. Compared to the control cells, A549 cells transfected with si‐PSPH exhibited significantly decreased migration and invasion capacities by Transwell and wound‐healing assays, respectively (Fig 5). These results indicate that PSPH knockdown may decrease the migratory and invasive potential of human NSCLC cells.

### PSPH regulates the AKT/AMPK signaling pathway in NSCLC cells

**Figure 6:**
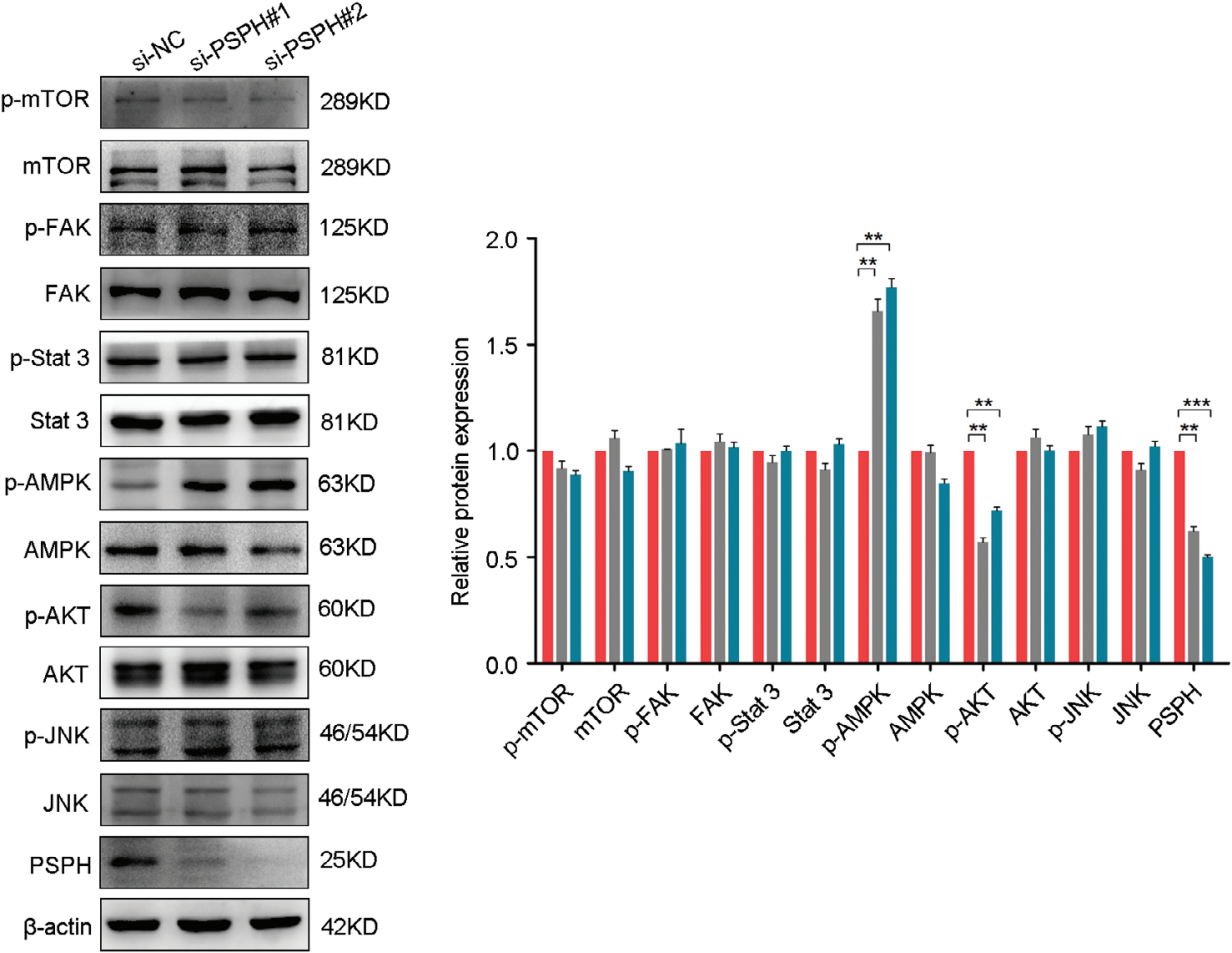
Phosphoserine phosphatase (PSPH) regulates the AKT/AMPK signaling pathway. Western blot analysis of p‐mTOR, mTOR, p‐FAK, FAK, p‐Stat3, Stat3, p‐AMPK, AMPK, p‐AKT, AKT, p‐JNK, JNK, and PSPH in A549 cells transfected with small interfering negative control (si‐NC) and si‐PSPH. (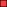) si‐NC, (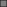) si‐PSPH#1, and (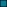) si‐PSPH#2.

To explore the possible downstream signaling pathways by which PSPH modulates the progression of NSCLC cells, we investigated the effects of PSPH on cell survival, proliferation, invasion, and metastasis‐related signaling molecules in A549 cells after si‐PSPH transfection, including mTOR, FAK, Stat3, AMPK, AKT, and JNK. Once phosphorylated, proteins can exert their function, while the forms of total proteins have no function. Therefore, we examined whether PSPH could regulate phosphorylated protein levels downstream of these pathways. si‐PSPH suppressed the phosphorylation of AKT and promoted the phosphorylation of AMPK compared to the si‐NC cohort in the A549 cell line, but had no effect on p‐mTOR, p‐FAK, p‐STAT3, or p‐JNK (Fig 6). No change was observed in the total protein levels of any of these molecules. These data indicate that PSPH may potentially facilitate the migration, invasion, and proliferation of NSCLC cells by regulating the AKT/AMPK signaling pathway.

## Discussion

Lung cancer is one of the most frequently occurring cancers, resulting in the highest mortality rate among all cancers.^16^ Metastasis, one of the most critical hallmarks of cancer, is the leading cause of NSCLC‐related death worldwide.^17^ Therefore, there is an urgent need to identify the potential therapeutic targets and understand the underlying molecular mechanisms of NSCLC.

Previous studies have shown that high PSPH expression in many cancers is significantly related to poor prognosis, positive metastasis, and high clinicopathologic stage, including colorectal cancer, phyllodes tumor,^11^ breast cancer,^12^ Ewing sarcoma,^14^ and hepatocellular carcinoma.^18^ For example, PSPH contributes to cell survival and proliferation by interacting with c‐MYC under glucose/glutamine deprivation conditions in hepatocellular carcinoma.^18^ Additionally, a high PSPH level promotes cancer cell proliferation and survival through an accumulation of reactive oxygen species and DNA damage, and facilitates brain and bone metastasis by simulating osteoclastogenesis.^19^, ^20^, ^21^ These findings demonstrate that PSPH may play a vital role in tumorigenesis and progression and may serve as a promising molecular target for human cancer.

Of note, the *PSPH* gene is located close to *EGFR* on chromosome 7 (positions 7p11.2 and 7p12, respectively), a frequent chromosome amplification region observed in NSCLC. However, the prognostic role of PSPH in NSCLC patients has never been investigated. In this study, we observed higher PSPH expression in NSCLC tissues than in corresponding non‐cancerous tissues. Furthermore, PSPH overexpression was positively related with lymph node or distant metastasis and malignant phenotype. Statistical analyses suggested that PSPH overexpression predicts poor survival in patients with NSCLC. In addition, we observed that PSPH knockdown inhibited NSCLC cell migration, invasion, and proliferation by inducing cell cycle arrest in the G2/M phase. Thus, our results show that PSPH could be a potential therapeutic target in NSCLC.

Previous studies have demonstrated that AKT/AMPK signaling plays a pivotal role in a wide range of essential processes for development and progression, including cell survival, proliferation, apoptosis, and metastasis.^22^, ^23^ However, its relationship with other molecular alterations observed in NSCLC has not been clearly elucidated. Our results show that PSPH knockdown could inhibit AKT phosphorylation and promote AMPK phosphorylation. We speculated that PSPH‐mediated NSCLC cell progression and aggressiveness might be at least partly attributed to the activation of the AKT/AMPK signaling pathway, which is critical for the initiation and progression of NSCLC. However, the direct regulation relationship between PSPH and the AKT/AMPK signaling pathway requires further investigation in future studies.

In conclusion, our study provides insight into the clinical significance of PSPH in NSCLC and demonstrates that PSPH activates the AKT/AMPK signaling pathway, leading to enhanced NSCLC cell proliferation, migration, and invasion. On the basis of this data, we speculate that PSPH may play an important role in NSCLC progression and aggressiveness through the AKT/AMPK signaling pathway, and thus may be a novel therapeutic target for NSCLC.

## Disclosure

No authors report any conflict of interest.
